# Evaluation of the GeneXpert for Human Monkeypox Diagnosis

**DOI:** 10.4269/ajtmh.16-0567

**Published:** 2017-02-08

**Authors:** Daniel Li, Kimberly Wilkins, Andrea M. McCollum, Lynda Osadebe, Joelle Kabamba, Beatrice Nguete, Toutou Likafi, Marcel Pie Balilo, Robert Shongo Lushima, Jean Malekani, Inger K. Damon, Michael C. L. Vickery, Elisabeth Pukuta, Frida Nkawa, Stomy Karhemere, Jean-Jacques Muyembe Tamfum, Emile Wemakoy Okitolonda, Yu Li, Mary G. Reynolds

**Affiliations:** 1 Poxvirus and Rabies Branch, Division of High-Consequence Pathogens and Pathology, National Center for Enteric and Zoonotic Infectious Diseases, Centers for Disease Control and Prevention, Atlanta, Georgia.; 2 Kinshasa School of Public Health, Kinshasa, Democratic Republic of Congo.; 3 Centers for Disease Control and Prevention, Kinshasa, Democratic Republic of Congo.; 4 University of Kinshasa, Kinshasa, Democratic Republic of Congo.; 5 BioGX, Birmingham, Alabama.; 6 Institut National de Recherche Biomédicale, Ministry of Health, Kinshasa, Democratic Republic of Congo.; 7 Ministry of Health, Kinshasa, Democratic Republic of Congo.

## Abstract

Monkeypox virus (MPXV), a zoonotic orthopoxvirus (OPX), is endemic in the Democratic Republic of Congo (DRC). Currently, diagnostic assays for human monkeypox (MPX) focus on real-time quantitative polymerase chain reaction (PCR) assays, which are typically performed in sophisticated laboratory settings. Herein, we evaluated the accuracy and utility of a multiplex MPX assay using the GeneXpert platform, a portable rapid diagnostic device that may serve as a point-of-care test to diagnose infections in endemic areas. The multiplex MPX/OPX assay includes a MPX-specific PCR test, OPX-generic PCR test, and an internal control PCR test. In total, 164 diagnostic specimens (50 crusts and 114 vesicular swabs) were collected from suspected MPX cases in Tshuapa Province, DRC, under national surveillance guidelines. The specimens were tested with the GeneXpert MPX/OPX assay and an OPX PCR assay at the Institut National de Recherche Biomedicale (INRB) in Kinshasa. Aliquots of each specimen were tested in parallel with a MPX-specific PCR assay at the Centers for Disease Control and Prevention. The results of the MPX PCR were used as the gold standard for all analyses. The GeneXpert MPX/OPX assay performed at INRB had a sensitivity of 98.8% and specificity of 100%. The GeneXpert assay performed well with both crust and vesicle samples. The GeneXpert MPX/OPX test incorporates a simple methodology that performs well in both laboratory and field conditions, suggesting its viability as a diagnostic platform that may expand and expedite current MPX detection capabilities.

## Introduction

Monkeypox virus (MPXV) is a zoonotic orthopoxvirus (OPX) endemic to west and central Africa. Each year, majority of human monkeypox (MPX) infections are reported from the Congo Basin in the Democratic Republic of Congo (DRC).^1^,^2^ Since 2005, laboratory-confirmed cases of MPX have also been reported from Republic of the Congo, Central African Republic, Sudan,^3^–^5^ Sierra Leone (unpublished data, US Centers for Disease Control and Prevention), and Cameroon (unpublished data, World Organization of Animal Health). Serologic evidence of OPX infection in Sierra Leone suggests human exposure to wild-type zoonotic OPXs in west Africa.^6^ Although smallpox vaccination with Vaccinia virus provides protection against human MPXV infection,^7^ lack of routine vaccination after smallpox eradication in 1980 has been hypothesized to have contributed to an increase in human MPX incidence.^8^,^9^

MPXV is transmitted to humans from a primary zoonotic source, and the virus is also capable of human-to-human transmission.^10^ The animal reservoir of MPXV remains unknown; however, several genera of African rodents (*Cricetomys*, *Graphiurus*, *Funiscirurus*) have been identified as potential reservoirs responsible for transmission and maintenance of the virus.^11^,^12^ Contact with wildlife, which often occurs in rural communities in DRC that are dependent on bushmeat as a protein source, is thought to increase risk for human infection with MPXV.^13^

Human infection with MPXV can lead to a smallpox-like illness with an 11% fatality rate in unvaccinated individuals.^14^ Upon infection, the estimated incubation period is on average 7–14 days, but can range from 5 to 21 days. The disease is characterized by a prodrome of fever, chills, headache, and myalgia lasting 1–3 days, followed by a maculopapular eruption. The median of number of days from onset of fever to onset of rash is about 2 days. The rash progresses slowly over a 2- to 3-week period, evolving through vesicular and pustular stages, and culminating in crusts.^14^–^17^ At the earliest stages of illness—prior to advanced rash lesion differentiation—MPX can be difficult to discriminate from other rash illnesses such as varicella.^18^ In DRC, over 2,000 cases of suspected MPX are reported to the Ministry of Health annually, but only a subset of those cases are formally investigated with laboratory confirmation of diagnostic specimens.

Molecular diagnostics for human MPX are currently limited to real-time quantitative polymerase chain reaction (qPCR) assays in specialized laboratory settings.^19^ Delayed clinical diagnosis and lack of etiologic confirmation (via laboratory testing) are impediments to controlling the spread of MPX. Increased diagnostic capabilities in areas at risk for MPX would allow for an efficient and timely public health response in the case of an outbreak. Therefore, a relatively simple system designed to perform well in less sophisticated laboratories and field conditions would aid in surveillance and disease control activities.

The Cepheid GeneXpert system is a backpack-sized, consolidated analytic workstation that combines sample preparation with real-time PCR amplification and detection. The system uses a self-contained cartridge to minimize contamination risks, and results are obtained from minimally processed samples in less than 90 minutes. This technology has been used for the detection of a number of infectious diseases, most notably *Mycobacterium tuberculosis* with rifampicin resistance, methicillin-resistant *Staphylococcus aureus*, and, most recently, Ebola virus disease (EVD).^20^–^22^ In this study, we evaluated the accuracy and utility of a multiplex MPXV and OPX assay using the GeneXpert platform, to provide an alternative to traditional PCR detection methods.

## Methods

### Surveillance methods and specimen collection.

In 2010, the U.S. Centers for Disease Control and Prevention (CDC) and the Kinshasa School of Public Health (KSPH) began a program with the DRC Ministry of Health to enhance surveillance for human MPX in Tshuapa Province (formerly Tshuapa District in Equateur Province). The population of Tshuapa Province is approximately 1.6 million persons, and 600–900 cases of suspected MPX are reported from the province each year. Procedures for enhanced surveillance included completion of an MPX-specific case investigation form and collection of at least one diagnostic specimen from ill persons who met the clinical criteria specified in the suspected MPX surveillance case definition—an illness characterized by vesicular pustular eruption with well-circumscribed, deep-seated lesions and at least one of the following symptoms: fever preceding the eruption, lymphadenopathy (inguinal, axillary, and/or cervical), and/or pustules or crusts on the palms of the hands or soles of the feet.

**Figure 1.**
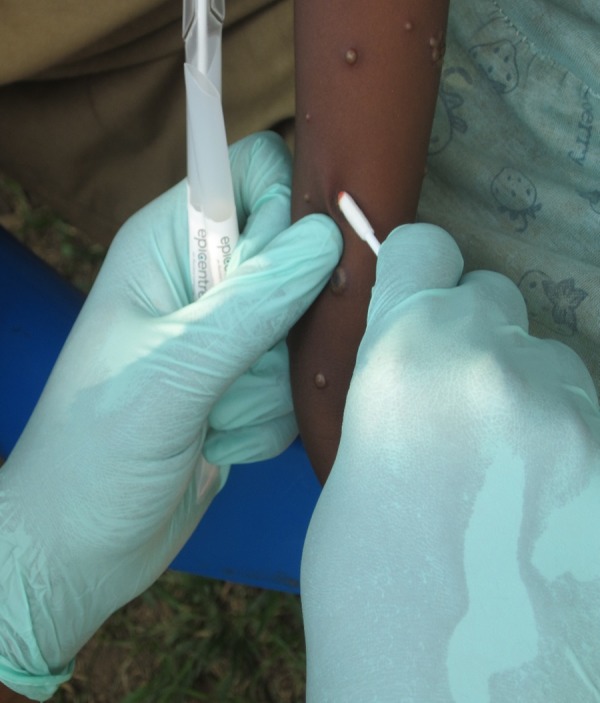
Unroofing of a pustular lesion during specimen collecion (photo credit: A. McCollum).

Lesion specimens collected for diagnosis were 1) vesicular or pustular lesion fluid, collected by touching the tip of a sterile Dacron swab to the lesion; 2) lesion crusts, collected by using the blunt end of a sterile tool to dislodge a crust; and/or 3) vesicular or pustular lesion roof, collected using the blunt end of the sterile tool to dislodge the friable outer surface of the lesion (Figure 1

). All specimens were stored in a sterile tube at 4°C. Specimens and case investigation forms were transferred to the national laboratory, Institut National de Recherche Biomedicale (INRB), in Kinshasa.

As an addendum to routine testing, patients (or family proxy) were asked by Ministry of Health–designated surveillance personnel to consent to having limited additional testing performed on the diagnostic specimens. This study received ethical approval from institutional review boards at the CDC and KSPH.

### Specimen preparation.

Crust and vesicular swab specimens were processed for testing and storage at INRB.

Swab specimens were hydrated in 1.5 mL of phosphate-buffered saline (PBS) and then spun on a benchtop microcentrifuge for 30 seconds, after which the supernatant was eluted. Crust specimens were first homogenized in 0.5 mL of PBS solution, then an additional 1.0 mL of PBS was added, and the sample was spun on a benchtop centrifuge for 30 seconds to settle particulate matter. For each sample type, approximately 1.5 mL of supernatant was produced.

For each specimen, 100 μL of supernatant was used for the MPX/OPX GeneXpert assay and 100 μL was used for DNA extraction (Qiagen EZ1 DNA Tissue Kit, Germantown, MD) and real-time OPX PCR at INRB.^23^ Additionally, 800 μL of the supernatant from each specimen was sent to the CDC for independent testing and validation, where 100 μL was used for a duplicate GeneXpert assay and 100 μL was used for DNA extraction and real-time MPX-generic PCR. At CDC, DNA was extracted from the supernatant using the EZ1 DNA Tissue Kit (Qiagen) in conjunction with the EZ1 platform (Qiagen).

All steps requiring specimen manipulation, including swab processing, crust homogenization, elution in PBS, and transfer to the GeneXpert cartridge were conducted within a certified biosafety cabinet.

### MPX-generic real-time PCR assay (gold standard).

Determination of the presence of MPXV DNA was accomplished using a real-time MPX-“generic” PCR assay with primers and fluorescent probes specifically designed for MPXV detection. The assay targeted the G2R region within the tumor necrosis factor receptor gene, and was capable of detecting all known strains of MPXV (hence, the use of the term “generic” to describe the assay). The assay was performed as previously described on the ABI 7500 DX Fast Real-Time PCR Instrument (Applied Biosystems, Foster City, CA) for 40 cycles, and specimens were run in duplicate.^24^ Results were assessed using existing clinical guidelines,^24^ which provided threshold cycle (Ct) value cutoffs for positive (< 37), indeterminate (37–39), and negative test results (0 or 40). Because indeterminate results did not result in a positive diagnosis, they were grouped with the negative results for the analyses in this study.

### GeneXpert MPX/OPX assay.

The GeneXpert MPX/OPX assay uses specialized cartridges for sample preparation and multiplex real-time PCR. The reagents were prepared beforehand in the form of lyophilized cakes and were packaged within the GeneXpert cartridge. This MPX/OPX assay was developed collaboratively with BioGX (Birmingham, Alabama), who aided in custom lyophilized real-time PCR reagent manufacturing, packaging of the real-time PCR reagents into the GeneXpert cartridges, design and performance optimization of the multiplex assay, and development and programing of the GeneXpert extraction and run protocols.

The primers for the MPX and OPX assays, G2R_G and E9L NVAR, respectively, were used as previously described.^24^,^25^ The limit of detection for the OPX assay was previously determined to be 2.56 fg of DNA or 12.5 genome copies on another platform.^25^ The assay utilized fluorescent labeled TaqMan^®^ style nuclease probes for the detection of the amplified targets. The probes used in the assay were labeled as follows: 6-carboxyfluorescein for the detection of MPXV, CalFluor Red 610 for the detection of OPX, and Cy5 for the detection of the internal amplification control (IAC). The three fluorophores have sufficient separation in both their excitation and emission spectra to allow for efficient multiplexing on the GeneXpert platform.

The MPX/OPX multiplex assay utilizes a spiked IAC to mitigate the false-negative reporting which could occur due to extraction of PCR-inhibiting substances from the sample matrix. The IAC contains synthesized DNA fragments that are not similar to known DNA sequences of any living organism; the IAC is a proprietary component of the cartridge. The IAC is an important reference for interpretation of negative and indeterminate results. The IAC template is limited to a low, but reliably amplifiable level, to allow efficient amplification with minimal effect on the reporting of the MPX and OPX targets in positive samples.

For sample preparation, 100 μL of the supernatant was combined with 600 μL of PBS and mixed before it was transferred to the sample-loading cartridge chamber. In addition, 1 mL of distilled water was added to a water-loading cartridge chamber. At INRB, cartridges were loaded onto a GeneXpert IV (Cepheid, Sunnyvale, CA) and operated according to manufacturer's instructions. At the CDC, either a GeneXpert IV or GeneXpert II platform was used.

Results for the MPX- and OPX-specific assays were assessed using the same criteria and Ct value cutoffs for clinical diagnosis as the MPX-generic real-time PCR assay.^24^ The possible assay outcomes and interpretation of results are listed in Table 1.

### Protocol change.

During the first round of specimen testing (November 2012–May 2013), it was found that the GeneXpert platform repeatedly experienced failed runs when assays were performed on crust samples. The error message displayed “Syringe pressure of *n* PSI exceeds the protocol limit of *m* PSI” (*n* and *m* referring to software pressure values). We hypothesized that, due to the particulate matter generated from pulverization of crust samples, the needle in the GeneXpert instrument became clogged, resulting in failed runs due to excessive syringe pressure. In February 2014, several changes were made during initial specimen preparation steps performed at INRB (as described above). An extra crust homogenization step was added, as well as an extra centrifugation step to decrease particulate matter. The results from both before and after the protocol change were analyzed; crust specimens that produced an error message were not subjected to a new preparation and reanalyzed.

### Data analysis.

The goal of this study was not only to compare the performance of the GeneXpert platform to the PCR gold standard, but also to ensure that the GeneXpert compared well under natural conditions at the national laboratory in DRC. Therefore, we compared GeneXpert results from both the CDC and INRB to the results of the MPX-generic real-time PCR assay run at the CDC. Finally, for a relative comparison to existing capabilities, we also compared the results of the INRB OPX PCR assay results to those of the gold standard.

All data analyses were conducted using SPSS version 21.0 (Chicago, IL).

## Results

### Characteristics of specimens.

In total, 164 specimens were collected from 161 suspected MPX cases (79 males and 82 females). The median age of patients was 16 years (range was 0 to 79 years); 72 specimens (45%) were collected from adults, and 89 (55%) were collected from children (< 18 years). Of these specimens, 50 (30%) were crusts and 114 (70%) were vesicular swabs (Table 2).

Eighty-eight (55%) specimens were positive for the presence of MPXV DNA signatures via MPX-generic real-time PCR assay conducted at the CDC. An additional 60 (37%) specimens were negative, and 13 (8%) yielded indeterminate results. Combining the negative and indeterminate categories, 73 (45%) specimens were classified as negative for purposes of the analyses that follow.

### Specimen timeline.

Specimens were collected from patients with active illness through the enhanced disease surveillance system. The patients who participated in this study reported developing a rash after a mean of 2.5 days (range was −7 to 33 days) post-illness onset. Patients received care from a health-care worker after an average of 5.8 days post-rash onset (range was −6 to 30 days), at which time a case investigation was performed and specimens were collected. After specimen collection, it took an average of 44.1 days (range, 10 to 207 days) for the specimen to be received at the national laboratory in Kinshasa. Sixty-five percent of specimens were tested 1 month or longer after specimen collection and about 10% of the specimens were tested 2 months or greater after specimen collection. There were no differences in MPX-positive versus negative specimens between the average time of collection to specimen arrival in Kinshasa (45.98 days versus 41.96 days [two-sample *t*-test, *P* value = 0.117], respectively) (Table 3).

### Performance of the GeneXpert MPX/OPX assay.

When compared with the MPX-generic real-time PCR assay gold standard, the sensitivity of the GeneXpert MPX/OPX test conducted at INRB was 98.8% (83/84) and the specificity was 100% (65/65). The positive predictive value (PPV) was 100% (83/83) and negative predictive value (NPV) was 98.5% (64/66). The sensitivity of the GeneXpert MPX/OPX test conducted at CDC was 98.6% (74/75) and the specificity was 100% (71/71). The PPV for the CDC GeneXpert MPX/OPX test was 100% (74/74) and the NPV was 98.6% (71/72).

With respect to specimen type, crusts and vesicles performed similarly on the GeneXpert platform at both INRB and CDC (Table 4). For example, crust samples tested at the INRB yielded a sensitivity of 100% (23/23) and a specificity of 100% (20/20), whereas vesicular swabs gave a sensitivity of 98.4% (60/61) and a specificity of 100% (45/45).

During the first round of specimen testing (November 2012–May 2013), the GeneXpert platform experienced difficulties in processing crust samples: 23%(7/30) of crust specimens tested at the INRB and 47% (14/30) of those tested at the CDC demonstrated at least one failed run and required further testing. After protocol changes were instituted in February 2014, only 5% (7/134) of specimens tested at the INRB and 3% (4/134) of those tested at the CDC experienced failed runs.

At INRB, the standard for MPXV testing was a generic OPXV real-time PCR assay. When compared with the MPX-generic real-Time PCR assay gold standard, the OPXV assay had a sensitivity of 72.7% (64/88) and a specificity of 82.3% (51/62) (Table 4).

Ct values for specimens using the MPX-generic real-time PCR assay gold standard were compared with positive Ct values obtained from the GeneXpert platforms at INRB or CDC (from the same specimens). The mean, median, minimum, and maximum Ct values were comparable across platforms and instruments.

### MPX/OPX multiplex assay efficiency.

When multiplexing, the PCR efficiency can be negatively affected due to competition between the separate PCR tests in the multiplex, as well as, the increased consumption and sharing of reaction components compared with simplex reactions. Both the MPX and OPX assays were previously validated in simplex with efficiencies of 90% and 83%, respectively.^24^,^25^ In multiplex, the MPX assay had an efficiency of 95%, and OPX had an efficiency of 91% (data not shown); thus, efficiency was observed to increase when the simplex tests were multiplexed together.

## Discussion

The GeneXpert MPX/OPX assay displayed high sensitivity, specificity, NPV, and PPV in specimens from suspect cases. The assay was highly accurate regardless of the type of specimen collected (crust versus vesicular swab), the place of testing (INRB or CDC), and demonstrated the ability to identify MPX infection at any stage of the rash. Blood viremia after MPXV infection is often time dependent and unreliable for acute diagnosis,^5^ so crust and vesicular swab specimens were preferable, and also reduced the risks associated with needle use for the patients. Further, crusts and vesicular swabs were used for their reliability and stability with the GeneXpert cartridge.

The initial deployment of the GeneXpert platform required some initial trial and error to determine optimal cartridge preparation procedures to minimize the number of failed runs. The initial, relatively high rate of failed runs is hypothesized to have been due to the large amount of particulate matter generated during the pulverization of crusts, which could have clogged the needle instrument. The specimen processing procedures were modified slightly, which sharply decreased the number of failed runs. Training to troubleshoot and interpret GeneXpert protocols is necessary to optimize performance.

An important advantage of the GeneXpert system as a diagnostic platform for testing of specimens suspected of harboring OPXV is that only minimal sample manipulation is needed prior to loading the specimen into the cartridge. This both increases the safety for the person performing the test and reduces contamination risks, especially during DNA extraction steps. Although the closed reaction chamber significantly reduces contamination after the test cartridge has been prepared, potential contamination during specimen processing steps could lead to erroneous results. Surfaces and tools used to process specimens should be extensively cleaned and decontaminated to avoid contamination with foreign DNA. Furthermore, reducing the number and difficulty of steps for specimen preparation has the additional benefit of reducing the need for highly trained operators to conduct the test, though training in laboratory safety and molecular diagnostic techniques is still recommended.

In DRC, human MPX diagnostics are currently limited to real-time PCR in a specialized reference laboratory located in Kinshasa. In this study, the average time for a specimen to be delivered to the national laboratory after collection was about a month and a half. The current long turnaround time between specimen collection and testing hinders the ability to implement effective public health safety measures and infection control practices in the event of an MPX outbreak. In this study, the assay still performed well despite the large turnaround times, which is a testament to a combination of the stability of MPX specimens and robust performance parameters of the GeneXpert platform. However, positioning a GeneXpert machine closer to the point of case detection could accelerate diagnosis to within days of case detection, leading to quicker disease management, taking advantage of the safety and ease which allows for its ability of decentralized testing.

Currently, costs associated with the GeneXpert system and its cartridges may constitute a barrier to more widespread use. The cost for a GeneXpert machine—with four modules—is currently about USD 60,000 (two module, USD 40,000), and each MPX/OPX assay cartridge costs approximately USD 75. Compared with traditional real-time PCR, which costs about USD 5–10 per test, lower costs for the GeneXpert cartridges will be required for broader use of this test for routine diagnostics. However, multiplexing provides a way to reduce reaction costs by as much as 40%. The cost of a multiplex reaction is close to that of a singleplex reaction, so performing multiplex reactions offers savings when compared with performing three singleplex reactions independently, for example.

Another consideration for rural field settings is the need for a reliable power source for the GeneXpert. In Tshuapa Province, for example, power sources are limited, which would limit its use for bedside diagnosis. However, since the GeneXpert platform is being used for diagnostics for other infectious diseases, most notably EVD and tuberculosis, the ability to implement this assay where the technology is already being used may increase the feasibility of the test where human MPX and these other infectious diseases overlap. Newer versions with increased portability and battery life may also become available soon, overcoming some current limitations.

Overall, we found the GeneXpert MPX/OPX test to be a specific and accurate test for detecting human MPX infection. Compared with traditional real-time PCR diagnostic methods, it is not only easier and much faster to use, but also offers lower risk of contamination and increases user safety through its all-in-one cartridge system. This study highlights the importance of optimizing detection parameters for qPCR tests to create versatile, but specific multiplex assays that may succeed in both specialized laboratory environments and field conditions. Analytical results provided by the GeneXpert MPX/OPX test, in conjunction with other pertinent clinical and epidemiological data may provide a promising alternative to current laboratory diagnostic capabilities in diagnosing MPX infection in suspected cases.

## Figures and Tables

**Table 1:** Array of possible results using the GeneXpert multiplex assay for MPX detection*

GeneXpert test interpretation	MPX assay	OPX assay	IAC assay
MPX positive	Positive	Positive	Positive/negative*
MPX negative	Negative	Positive/negative	Positive/negative
OPX positive	Positive/negative	Positive	Positive/negative
MPX, OPX negative	Negative	Negative	Positive
Negative through inhibition	Negative	Negative	Negative

IAC = internal amplification control; MPX = monkeypox, OPX = orthopoxvirus.

* In multiplex assays, reaction components are shared competitively between separate polymerase chain reaction tests in the multiplex. The IAC template is limited to a low, but reliably amplifiable level to test for sample quality and inhibition for MPX- and OPX-negative cases. However, in samples of poor quality (demonstrated by high MPX and/or OPX assay Ct values), the IAC may amplify though still yielding a MPX- and/or OPX-positive test result.

**Table 2:** Characteristics among all cases and and confirmed monkeypox cases

	Total *N* = 161	Confirmed monkeypox cases* *N* = 88
Male	79	41
Female	82	47
Age		
< 18	89	41
≥ 18	72	47
Specimen type†		
Crust	50	25
Vesicular swab	114	63

* Cases with at least one specimen tested positive for monkeypox using a monkeypox-generic real-time polymerase chain reaction assay (gold standard) at the Centers for Disease Control and Prevention.

† A total of 164 specimens were collected from 161 cases.

**Table 3:** Timeline of MPX symptoms to specimen receipt at the National Laboratory (number of days)

	Fever to rash		Rash to specimen collection		Specimen collection to laboratory	
	MPX positive	MPX negative	MPX positive	MPX negative	MPX positive	MPX negative
Mean	2.2	2.9	5.4	6.0	46.0	42.0
Median	2.0	2.0	5.0	5.0	39.0	40.0
Range	−5.0 to 9.0	−7.0 to 33.0	−6.0 to 23.0	0.0 to 30.0	10.0 to 207.0	12.0 to 185.0

MPX = monkeypox.

**Table 4:** Performance of assays in relation to the MPX-generic real-time PCR assay (gold standard) by specimen type

	Crusts (*N* = 50)				Vesicles (*N* = 114)			
	Sensitivity (%)	Specificity (%)	PPV (%)	NPV (%)	Sensitivity (%)	Specificity (%)	PPV (%)	NPV (%)
INRB MPX/OPX* GeneXpert	100	100	100	100	98.4	100	100	97.8
CDC MPX/OPX GeneXpert	100	100	100	100	98.3	100	100	97.6
OPX PCR	68.0	92.0	89.5	74.2	74.6	85.4	79.7	68.6

CDC = Centers for Disease Control and Prevention; INRB = Institut National de Recherche Biomedicale; MPX = monkeypox; NPV = negative predictive value; OPX = orthopoxvirus; PCR = polymerase chain reaction; PPV = positive predictive value.

* MPX/OPX refers to the monkeypox/orthopoxvirus multiplex assay run on the GeneXpert platform.
